# Predictive value of the videofluoroscopic swallowing study for long-term mortality in patients with subacute stroke

**DOI:** 10.1097/MD.0000000000028623

**Published:** 2022-01-28

**Authors:** Daham Kim, Jae-Hyung Kim, Si-Woon Park, Hyung-Wook Han, Sang Joon An, Yeong In Kim, Hyo Jin Ju, YoonHee Choi, Doo Young Kim

**Affiliations:** a Department of Rehabilitation Medicine, College of Medicine, Catholic Kwandong University International St Mary's Hospital, Incheon, Korea; b Department of Neurology, College of Medicine, Catholic Kwandong University, International St. Mary's Hospital, Incheon, Korea; c Department of Medical Humanities, College of Medicine, Catholic Kwandong University, Gangwon, Korea; d The Convergence Institute of Healthcare and Medical Science, College of Medicine, Catholic Kwandong University, Incheon, Korea.

**Keywords:** deglutition disorder, diet, mortality, rehabilitation, stroke

## Abstract

To investigate the usefulness of the videofluoroscopic swallowing study (VFSS) for subacute stroke in predicting long-term all-cause mortality, including not only simple parameters obtained from VFSS results, but also recommended dietary type as an integrated parameter.

This was a retrospective study of patients with subacute (<1 month) stroke at a university hospital between February 2014 and September 2019. The independent risk factors were investigated using stepwise Cox regression analysis, which increased the all-cause mortality of patients with stroke among VFSS parameters.

A total of 242 patients with subacute stroke were enrolled. The significant mortality-associated factors were age, history of cancer, recommended dietary type (modified dysphagia diet; adjusted hazard ratio [HR], 6.971; *P* = .014; tube diet, adjusted HR: 10.169; *P* = .019), and Modified Barthel Index. In the subgroup survival analysis of the modified dysphagia diet group (*n* = 173), the parameters for fluid penetration (adjusted HR: 1.911; 95% confidence interval, 1.086-3.363; *P* = .025) and fluid aspiration (adjusted HR: 2.236; 95% confidence interval, 1.274-3.927; *P* = .005) were significantly associated with mortality.

The recommended dietary type determined after VFSS in subacute stroke was a significant risk factor for all-cause mortality as an integrated parameter for dysphagia. Among the VFSS parameters, fluid penetration and aspiration were important risk factors for all-cause mortality in patients with moderate dysphagia after stroke. Therefore, it is important to classify the degree of dysphagia by performing the VFSS test in the subacute period of stroke and to determine the appropriate diet and rehabilitation intervention for mortality-related prognosis.

## 1 Introduction

As the older population increases, pneumonia is becoming one of the most common causes of death.^[1]^ Several studies have shown that between 50% and 60% of community-acquired pneumonia cases are aspiration pneumonia.^[2–4]^ In addition, 30% to 65% of patients with stroke suffer from dysphagia, and 14.3% suffer from aspiration pneumonia.^[5–7]^ Poststroke dysphagia increases the risk of aspiration pneumonia from 3 to 11 times, and is an independent risk factor that increases all-cause mortality regardless of the type of stroke.^[8,9]^ In one study, stroke patients with dysphagia had 1.84 times higher all-cause mortality rate than those with stroke without dysphagia.^[9]^ It is well known and theoretically accepted that dysphagia increases the all-cause mortality rate of patients with stroke.

The videofluoroscopic swallowing study (VFSS) is a gold standard that verifies swallowing function, and it is a naturally accepted concept that the results of VFSS may be related to long-term mortality in patients with stroke.^[10–13]^ Through the VFSS test, the following simple parameters related to swallowing function were obtained: aspiration or penetration, residuals after swallowing, transit time through swallowing, laryngeal elevation, and other variable parameters associated with swallowing.^[14]^ Physicians integrate all objective simple parameters and recommend the most appropriate dietary type and rehabilitation intervention to avoid aspiration.^[15]^ However, some studies report that the aspiration or penetration found in videofluoroscopy performed in patients with acute stroke is not related to the long-term mortality rate of patients and insist that the value of detecting aspiration through the VFSS test is questionable.^[16–18]^

In this study, we assumed that the recommended dietary type is an integrated parameter of dysphagia severity concluded by comprehensively integrating the patient's condition, such as physical examination and medical history, as well as objective simple VFSS parameters. We hypothesized that the first recommended dietary type, as an integrated parameter indicating swallowing function, may be associated with long-term all-cause mortality of subacute stroke patients. We also hypothesized that the effect of simple VFSS parameters on all-cause mortality may differ according to the degree of dysphagia of subacute stroke patients. The purpose of this study was to investigate the usefulness of the VFSS for subacute stroke in predicting long-term all-cause mortality, including not only simple parameters obtained from VFSS results, but also recommended dietary type as an integrated parameter.

## 2 Participants and methods

### 2.1 Participants

Patients with subacute (<1 month) stroke who underwent VFSS at a university-affiliated hospital between February 2014 and September 2019 were studied retrospectively. The survival survey was conducted on May 20, 2020. In our hospital setting, all acute stroke patients with an initial National Institute of Health Stroke Scale score of 3 or higher at admission were referred to the Department of Rehabilitation Medicine.^[19]^ The referred patients were evaluated comprehensively for motor function, cognitive function, activities of daily living, and swallowing function. All patients with stroke referred to the Department of Rehabilitation Medicine were assessed via VFSS for swallowing function, except for patients with severe severity and difficulty maintaining a wheelchair sitting position. In this study, patients who underwent VFSS within 1 month from onset were enrolled through a retrospective review of the medical record data of stroke patients who proceeded with the treatment process.

**Figure 1:**
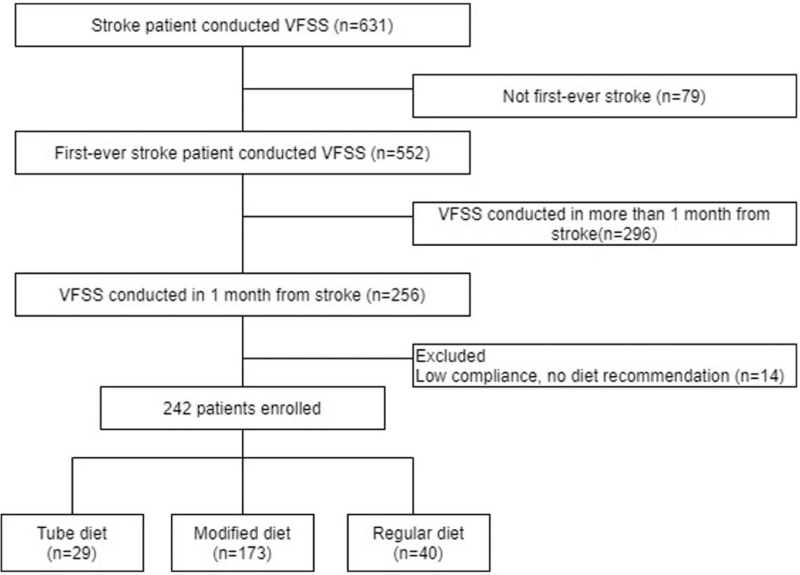
Flowchart of subject enrolment of this study. The enrolment of subjects was performed retrospectively. A total of 631 stroke patients underwent for VFSS. Based on the exclusion criteria, 242 patients were enrolled. Twenty-nine patients were prescribed a tube diet. A modified dysphagia diet was prescribed to 173 patients. Forty patients were prescribed a regular diet. VFSS = videofluoroscopic swallowing study.

The inclusion criteria were as follows: patients who had first-ever stroke, both ischemic and hemorrhagic, and patients who had VFSS in 1 month after the stroke attack. Patients who performed the test but did not complete the examination due to poor cooperation were excluded. From the 256 patients from 1 month of stroke onset, 14 patients with low compliance were excluded. A total of 242 patients were enrolled in this study. The remaining 242 patients were categorized according to their dietary recommendations (tube diet group, modified dysphagia diet group, and regular diet group) (Fig. 1). Ethics approval for the conduct of the research was obtained from the Catholic Kwandong university hospital's Institutional Review Board (Serial Number: IS19RISI0065) on December 10, 2019. As this study was conducted using retrospective medical records, the requirement for informed consent was waived by the IRB.

### 2.2 VFSS protocol

Prior to the VFSS, we assessed the gag-reflex, 3-step obey test, and coughing reflex. VFSS was performed according to a modified version of Jeffrey's protocol.^[20]^ Using the International Dysphagia Diet Standardization Initiative framework, a spoon of level 3, level 5, and level 7 meals were provided sequentially, after which 2 mL, 6 mL, and 50 mL of fluid (level 0) were administered sequentially.^[21]^ Barium was mixed in all meals. The studies were performed using videofluoroscopy and recorded at 30 frames per second. A 24 mm diameter radiopaque coin was placed posterior to the neck for calibration during the analysis.

The recorded VFSS was analyzed by an experienced physiatrist who had been trained for more than 5 years. The oral phase was evaluated for lip closure, bolus formation, mastication function, tongue-palate contact, piecemeal swallowing, premature and bolus loss, and oral transit time. In the pharyngeal phase, pharyngeal reflex time, laryngeal elevation, completion of laryngeal closure, barium coating of the larynx wall after repeated trials, and residual volume and pharyngeal transit time were evaluated.

### 2.3 Simple parameters of VFSS

The penetration-aspiration scale (PAS) score was graded from 1 to 8 points.^[22]^ Score 1 indicates “material does not enter airway”. Score 2 points out “material enters the airway, remains above the vocal folds, and is ejected from the airway”. Score 3 means “material enters the airway, remains above the vocal folds, and is not ejected from the airway”. Score 4 is a sign of “material entering the airway, contacts the vocal folds, and is ejected from the airway”. A score of 5 indicates that the “material enters the airway, contacts the vocal folds, and is not ejected from the airway”. A score of 6 indicates that the “material enters the airway, passes below the vocal folds, and is ejected into the larynx or out of the airway”. Score 7 is “material enters the airway, passes below the vocal folds, and is not ejected from the trachea despite effort”. Lastly, score 8 means “material enters the airway, passes below the vocal folds, and no effort is made to eject”. In this study, penetration from PAS 2 to PAS 5 and aspiration from PAS 6 to PAS 8 were defined as per a previous study.^[23]^

Remnants of the pharynx are categorized as remnants of the vallecular space and the remnant of the piriform sinus using the videofluoroscopic dysphagia scale scoring system.^[24]^ Each space was classified into 3 categories based on the amount of remaining residue: mild (<10%), moderate (10%-50%), and severe (>50%).

### 2.4 Recommended dietary types

Recommended dietary types were determined according to the International Dysphagia Diet Standardization Initiative framework and graded using the Functional Oral Intake Scale.^[25,26]^

In our study, dietary types were classified into the following 3 types: tube feeding, modified dysphagia diet, and regular diet.

### 2.5 Data collection

Biometric data such as age and sex were collected. The type of stroke, survival month, and history of previous comorbidities such as diabetes mellitus, hypertension, cancer, and myocardial infarction were assessed. We investigated the Mini Mental State Examination and Modified Barthel Index (MBI) at the time of referral to assess the severity of stroke.^[27,28]^

The all-cause mortality surveillance of each patient was conducted using the National Health Insurance Service database in Korea.^[29]^

### 2.6 Statistical evaluation

Statistical analyses were performed using SPSS (version 22.0; IBM Corp., Armonk, NY). All patients were sorted into 3 groups according to the recommended dietary type: tube feeding, modified dysphagia diet, and regular diet. The differences among the 3 groups were assessed using one-way analysis of variance tests for continuous variables, and a chi-square test or Fisher exact test with the post hoc Bonferroni test for categorical variables were used to compare the groups. Frequencies and percentages were used to characterized categorical variables. For normally distributed variables, the mean (standard deviation) was used to characterized continuous variables.^[9,30,31]^ Multivariate Cox regression analysis (stepwise method) was performed to investigate the risk variables for all-cause mortality while controlling for possible confounders: myocardial infarction history, cancer history, diabetes mellitus history, hypertension history, recommended dietary type, initial MBI, initial Mini Mental State Examination score, stroke location, stroke type, and simple parameters of VFSS, such as remnant of vallecular space, remnant of the piriform sinus, aspiration, and penetration of each diet. The above confounders were known to be related to mortality of stroke patients.^[27,28,32–36]^ Whether the VFSS parameters are related to the mortality rate of stroke patients is not well known; therefore, we constructed a multivariate Cox regression model using a stepwise method in which only significant factors are automatically selected in the final model through a statistical method. A subgroup survival analysis was also performed for each recommended dietary group. Statistical significance was set at *P* < .05.

## 3 Results

### 3.1 General characteristics

A total of 242 patients were enrolled in this study. There were 127 (52.5%) men and 115 (47.5%) women.

The patients in the tube feeding group (n = 29), modified dysphagia diet group (n = 173), and regular diet group (n = 40), had a mean age of 72.1 ± 12.0 years, 67.9 ± 13.4 years, and 64.6 ± 13.9 years, respectively, and the mean age of all patients (n = 242) was 67.8 ± 13.4. The total number of deaths was 53 (21.9%), and the mean survival time was 848.5 ± 581.1 days. The all-cause mortality rates of each group were 31.0%, 24.3%, and 5.0%, respectively. The participants’ characteristics are presented in Table 1.

**Table 1:** General characteristics and comparison between each dietary group.

	Total (*n* = 242)	Regular (*n* = 40)	Modified (*n* = 173)	Tube (*n *= 29)	*P* value
Age (yrs)	67.8 ± 13.4	64.6 ± 13.9	67.9 ± 13.4	72.1 ± 12.0	.071
Sex, *n* (%)					.564
Female	115 (47.5%)	21 (52.5%)	79 (45.7%)	15 (51.7%)	
Male	127 (52.5%)	19 (47.5%)	94 (54.3%)	14 (48.3%)	
Initial MBI	35.8 ± 29.0	48.0 ± 28.2	35.3 ± 27.6	22.3 ± 32.2	.001^∗^
Initial MMSE	16.5 ± 9.2	19.5 ± 7.8	16.7 ± 9.2	11.2 ± 9.2	.001^∗^
Death, *n* (%)	53 (21.9%)	2 (5.0%)	42 (24.3%)	9 (31.0%)	.013^∗^
Survival time (d)	848.5 ± 581.1	1038.8 ± 609.8	809.1 ± 563.1	821.2 ± 614.8	.076
Location, *n* (%)					.484
Infratentorial	50 (20.6%)	7 (17.5%)	39 (22.5%)	4 (13.8%)	
Supratentorial	192 (79.4%)	33 (82.5%)	134 (77.5%)	25 (86.2%)	
Stroke type, *n* (%)					.085
Hemorrhage	55 (22.7%)	14 (35.0%)	37 (21.4%)	4 (13.8%)	
Infarction	187 (77.3%)	26 (65.0%)	136 (78.6%)	25 (86.2%)	
MI history, *n* (%)	2 (0.8%)	0 (0.0%)	1 (0.6%)	1 (3.4%)	.235
Cancer history, *n* (%)	5 (2.1%)	1 (2.5%)	4 (2.3%)	0 (0.0%)	.704
DM history, *n* (%)	82 (33.9%)	8 (20.0%)	62 (35.8%)	12 (41.4%)	.107
Hypertension history, *n* (%)	217 (89.7%)	37 (92.5%)	153 (88.4%)	27 (93.1%)	.607

Values, mean ± standard deviation. MBI = Modified Barthel Index, MMSE = Mini Mental State Examination, MI = myocardial infarction, DM = diabetes mellitus.

∗ *P* < .05.

### 3.2 Survival analysis

All-cause mortality was assessed using Cox regression analysis with age, sex, and other factors used to account for relevant confounders. Table 2 presents the results of univariate regression analysis.

**Table 2:** The relevance of mortality to each factor by using univariate regression analysis.

		95% CI		
	Hazard ratio	Lower	Upper	*P* value
Age	1.058	1.032	1.084	<.001^∗^
Sex	1.654	0.957	2.857	.071
MBI	0.986	0.975	0.996	.007^∗^
MMSE	0.970	0.943	0.997	.031^∗^
Hemorrhagic stroke (Ref; infarction)	0.323	0.138	0.758	.009^∗^
Supratentorial stroke (Ref; infratentorial)	0.735	0.358	1.508	.401
Aspiration or penetration of thick fluid (IDDSI 3)				.712
Penetration	1.265	0.631	2.538	.508
Aspiration	1.356	0.484	3.799	.563
Aspiration or penetration of soft meal (IDDSI 5)				.011^∗^
Penetration	2.062	1.046	4.065	.037^∗^
Aspiration	4.462	1.343	14.823	.015^∗^
Aspiration or penetration of regular meal (IDDSI 7)				.486
Penetration	1.158	0.485	2.764	.741
Aspiration	3.363	0.442	25.553	.241
Aspiration or penetration of fluid (IDDSI 0)				.225
Penetration	1.145	0.530	2.472	.730
Aspiration	1.735	0.879	3.424	.112
Recommended dietary type (Ref; regular diet)				.020^∗^
Modified dysphagia diet	6.325	1.529	26.156	.011^∗^
Tube diet	8.861	1.908	41.155	.005^∗^
Vallecular residue				.442
Mild (<10%)	1.449	0.329	6.387	.624
Moderate (10%-50%)	1.227	0.271	5.548	.790
Severe (>50%)	2.217	0.500	9.829	.295
Pyriform sinus residue				.767
Mild (<10%)	0.886	0.462	1.698	.716
Moderate (10%-50%)	1.301	0.561	3.013	.540
Severe (>50%)	1.503	0.454	4.981	.505
Myocardial infarction history	6.820	0.916	50.776	.061
Cancer history	7.815	2.791	21.881	<.001^∗^
Diabetes mellitus history	1.694	0.981	2.925	.058
Hypertension history	0.615	0.297	1.272	.190

CI = confidence interval, IDDSI = international dysphagia diet standardization initiative framework, MBI = Modified Barthel Index, MMSE = Mini Mental State Examination.

∗ *P* < .05.

Multivariate Cox regression analysis revealed that all-cause mortality-associated factors were age (adjusted hazard ratio [HR], 1.050; 95% confidence interval [CI], 1.021-1.080; *P* = .001), history of cancer (adjusted HR: 22.238; 95% CI, 6.623-74.666; *P* < .001), recommended dietary type (*P* = .040, modified dysphagia diet; adjusted HR: 6.971; 95% CI, 1.472-33.012; *P* = .014; tube diet; adjusted HR: 10.169; 95% CI, 1.462-70.723; *P* = .019), and MBI (adjusted HR: 0.987; 95% CI, 0.974-0.999; *P* = .037). However, penetration or aspiration of each diet, residuals of vallecular space and pyriform sinus, time from onset to VFSS, and sex were not significantly associated with mortality in patients with stroke (Table 3).

**Table 3:** The independent relevance of each factor to all-cause mortality investigated by using multivariate cox regression analysis including all subjects.

		95% CI		
	Adjusted hazard ratio	Lower	Upper	*P* value
Age	1.050	1.021	1.080	.001^∗^
Cancer history	22.238	6.623	74.666	<.001^∗^
Recommended dietary type	(Ref; regular diet)			.040^∗^
Modified dysphagia diet	6.971	1.472	33.012	.014^∗^
Tube diet	10.169	1.462	70.723	.019^∗^
MBI	0.987	0.974	0.999	.037^∗^

By the multivariate cox regression analysis, all confounders that were assessed are listed below, and only listed confounders were selected by stepwise selection. Assessed confounders: Age, sex, MBI, MMSE, hemorrhagic stroke (ref. infarction), supratentorial stroke (ref. infratentorial), aspiration or penetration of thick fluid (IDDSI 3), soft meal (IDDSI 5), regular meal (IDDSI 7), and fluid (IDDSI 0), recommended dietary type, vallecular residue, pyriform sinus residue, myocardial infarction history, cancer history, diabetes mellitus history, and hypertension history. CI = confidence interval, IDDSI = international dysphagia diet standardization initiative framework, MBI = Modified Barthel Index, MMSE = Mini Mental State Examination.

∗ *P* < .05.

**Figure 2:**
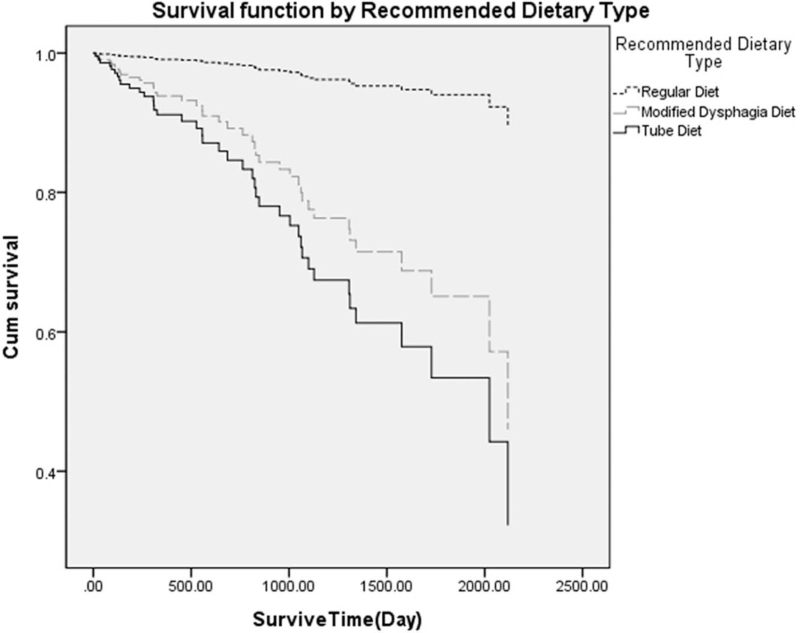
Cox proportional hazard model after VFSS in patients with stroke. The Cox proportional model showed that by the recommended dietary type, the tube diet group and the modified dysphagia diet group had a higher all-cause mortality rate than those with regular diet. VFSS = videofluoroscopic swallowing study.

A Cox proportional model was constructed according to the recommended dietary type, and it was confirmed that there was a difference in the mortality rate according to the recommended dietary type in stroke despite the statistical adjustment of confounding factors (Fig. 2). In this study, log minus log functions did not intersect each other, so it was appropriate to apply the Cox proportional hazard model.

### 3.3 Subgroup survival analysis

Subgroup analysis was additionally performed for each recommended dietary group using the covariate used in the multivariate Cox regression. There was no significant mortality associated factor among the VFSS parameters in the survival analysis of the tube diet group and the regular diet group.

In the subgroup survival analysis of the modified dysphagia diet group (*n* = 173), among the VFSS parameters, the parameters for the fluid penetration (adjusted HR: 1.911; 95% CI, 1.086-3.363; *P* = .025) and fluid aspiration (adjusted HR: 2.236; 95% CI, 1.274-3.927; *P* = .005) were significantly associated with long-term all-cause mortality (Table 4).

**Table 4:** The independent relevance of each factor to all-cause mortality investigated by using multivariate cox regression analysis including modified dysphagia diet subjects, as subgroup analysis.

		95% CI		
	Adjusted hazard ratio	Lower	Upper	*P* value
Aspiration or penetration of fluid (IDDSI 0)	(Ref; none)			.020^∗^
Penetration	1.911	1.086	3.363	.025^∗^
Aspiration	2.236	1.274	3.927	.005^∗^

By the multivariate cox regression analysis, all confounders that were assessed are listed below, and only listed confounders were selected by stepwise selection. Assessed confounders: Age, sex, MBI, MMSE, hemorrhagic stroke (ref. infarction), supratentorial stroke (ref. infratentorial), aspiration or penetration of thick fluid (IDDSI 3), soft meal (IDDSI 5), regular meal (IDDSI 7), and fluid (IDDSI 0), vallecular residue, pyriform sinus residue, myocardial infarction history, cancer history, diabetes mellitus history, and hypertension history. CI = confidence interval, IDDSI = international dysphagia diet standardization initiative framework, MBI = Modified Barthel Index, MMSE = Mini Mental State Examination.

∗ *P* < .05.

**Figure 3:**
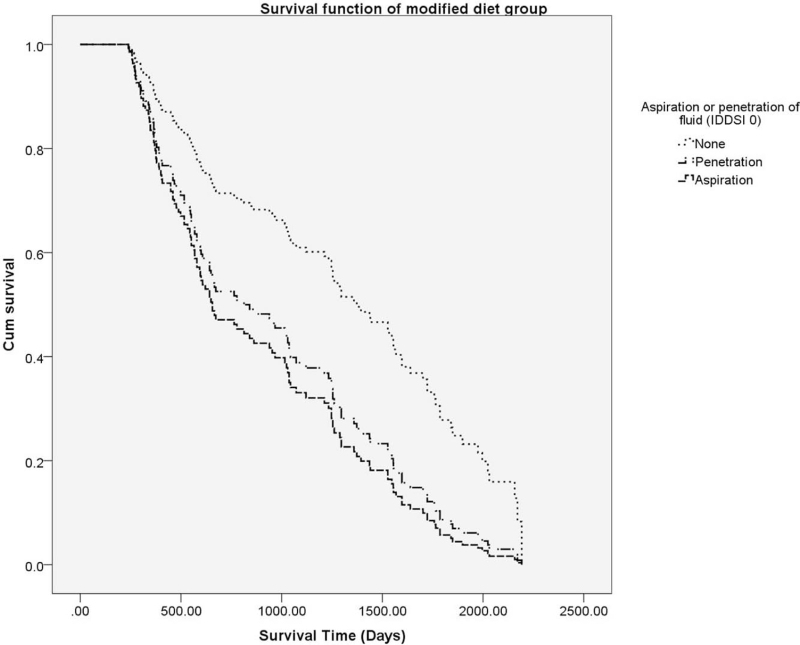
Cox proportional hazard model for the modified dysphagia diet group. In the modified dysphagia diet group, the Cox proportional model showed an increased mortality rate with fluid penetration and aspiration. IDDSI = international dysphagia diet standardization initiative framework.

According to the penetration and aspiration of the fluid, a Cox proportional model was constructed, and it was confirmed that there was a difference in the long-term mortality rate according to the penetration and aspiration of the fluid despite the statistical adjustment of confounding factors (Fig. 3). In this study, log minus log functions do not intersect with each other, so it is appropriate to apply the Cox proportional hazard model.

## 4 Discussion

Poststroke dysphagia is known to be a factor related to all-cause mortality, and the gold standard for diagnosing dysphagia is the VFSS. However, studies on the relationship between the outcomes of VFSS and mortality in stroke patients have reported inconsistent results.

In a previous study, Chen et al^[18]^ found no association between penetration and aspiration and mortality of subacute to chronic stroke patients. For this reason, there has been skepticism about the conduction of the VFSS test for subacute stroke patients. Yu et al^[37]^ showed that aspiration of VFSS can be a significant predictor of aspiration pneumonia for subacute and chronic stroke patients. Nativ-Zelter et al^[38]^ suggested that the mortality incidence was significantly higher in patients with dysphagia undergoing VFSS.^[12,16]^ In this study, as the analysis of all subjects who underwent VFSS within 1 month of stroke, simple parameters of VFSS, such as penetration and aspiration, were not associated with all-cause mortality but the recommended dietary type as an integrated parameter for dysphagia was associated with all-cause mortality. In the subgroup analysis, the impact of simple VFSS parameters on all-cause mortality differed according to the severity of dysphagia. In patients with severe dysphagia, the results of our study showed that VFSS parameters were not associated with all-cause mortality. We thought that this was because patients with severe dysphagia often had a higher overall stroke severity and higher mortality than from other causes, so the VFSS parameter had a reduced impact on overall mortality. In patients with no or mild dysphagia, the impact of VFSS parameters on overall mortality was also not significant, probably because mortality after mild stroke is rare in these patient groups. However, in our study, VFSS parameters were associated with mortality in the group with moderate dysphagia that received a modified diet recommendation. Therefore, it is necessary to classify the risk of dysphagia into large categories through the VFSS test in the subacute stroke period to identify risk groups and to provide appropriate dietary modification recommendations and therapeutic interventions.

Multivariate Cox regression analysis revealed that age, cancer history, recommended dietary type, and initial MBI were significantly associated with all-cause mortality in all patients with subacute stroke. Age and cancer history are well-known prognostic factors.^[39,40]^ Moreover, recent studies have shown that the activity of daily living, such as MBI, is also a significant prognostic factor for all-cause mortality of patients with stroke by indicating the severity of stroke.^[27,28,41,42]^ Aspiration and penetration on VFSS, as simple parameters, are well-known prognostic factors in patients with stroke.^[12,37]^ However, several studies have presented conflicting results indicating that the simple parameters of VFSS and mortality are not related.^[16,37]^ In our study, the simple parameter of VFSS was not associated with all-cause mortality, but the integrated parameter indicating the severity of dysphagia was associated with all-cause mortality of entire subacute stroke patients. The all-cause mortality rate was 6.971 times higher for those who were recommended modified dysphagia diets and 10.169 times higher for those who were recommended tube feeding, compared to those who were recommended a regular diet.

These findings show that the first recommended dietary type derived from VFSS can be used as an integrated prognostic factor for patients with subacute stroke.

In all subjects, the recommended dietary type, as an integrated parameter for dysphagia, had a significant relationship with all-cause mortality, but simple VFSS parameters showed no significant relationship with all-cause mortality. However, in situations with the same degree of dysphagia (for the same recommended diet group), simple VFSS parameters may be associated with mortality.

Since the impact of the simple VFSS parameters on all-cause mortality may differ from the severity of dysphagia, it is necessary to perform a subgroup analysis targeting groups with similar severity of dysphagia. Patients with severe dysphagia may have high overall stroke severity, resulting in increased all-cause mortality from other diseases, such as sepsis or internal organ failure. In contrast, in patients with no or minor dysphagia, the parameters associated with dysphagia may not affect all-cause mortality. The results of our study showed that simple VFSS parameters had no significant relationship with all-cause mortality in the severe and no or minor dysphagia groups. However, in the group with moderate dysphagia (modified dysphagia diet group), the all-cause mortality rate increased by 1.911 times with fluid penetration and 2.322 times with fluid aspiration, compared to normal results on the VFSS performed in the subacute period. These results support our hypothesis, indicating the importance of VFSS testing in the subacute period of stroke. In the subacute period of stroke, the overall severity of dysphagia was associated with all-cause mortality, and in patients with moderate dysphagia (modified dysphagia diet group), fluid penetration and aspiration, which were shown in the VFSS, were significantly associated with all-cause mortality. In our study, although aspiration and penetration were not associated with mortality in all subacute stroke patients, there was an association with mortality in patients with moderate dysphagia. The silent aspiration is sometimes referred to as aspiration without any apparent symptoms, and this can be confirmed through a VFSS test. Aspiration and penetration are factors that can be avoided or improved through the selection of an appropriate diet and intervention in rehabilitation treatment.^[12,13]^ Therefore, performing the VFSS test in the subacute period of stroke to classify the severity of dysphagia and determining the appropriate diet and rehabilitation intervention for them is important for their mortality-related prognosis.

The limitations of this study are as follows. First, the study had a retrospective design and a small sample size. Second, we were unable to define whether the factors were cause-specific to the disease because the causation of mortality was not investigated. In other words, the mortality rate in this study was limited to death due to the overall cause. In subsequent studies, it is necessary to confirm the association among the recommended dietary type, stroke severity, and mortality through a large-scale cohort study.

In conclusion, the recommended dietary type determined after VFSS in subacute stroke was a significant risk factor for all-cause mortality as an integrated parameter for dysphagia. Among the VFSS parameters, fluid penetration and aspiration were important risk factors for all-cause mortality in patients with moderate dysphagia after stroke. Therefore, it is important to classify the degree of dysphagia by performing the VFSS test in the subacute period of stroke and to determine the appropriate diet and rehabilitation intervention for mortality-related prognosis.

## Author contributions

**Conceptualization:** Doo Young Kim.

**Data curation:** Daham Kim, Doo Young Kim.

**Formal analysis:** Daham Kim, Si-Woon Park, Hyung-Wook Han, Hyo Jin Ju, YoonHee Choi, Doo Young Kim.

**Investigation:** Daham Kim, Hyung-Wook Han, Doo Young Kim.

**Methodology:** Sang Joon An, Yeong In Kim, Hyo Jin Ju, Doo Young Kim.

**Software:** Hyo Jin Ju, YoonHee Choi, Doo Young Kim.

**Supervision:** Jae-Hyung Kim, Si-Woon Park, Sang Joon An, Yeong In Kim, Hyo Jin Ju.

**Visualization:** Doo Young Kim.

**Writing – original draft:** Daham Kim, Doo Young Kim.

**Writing – review & editing:** Jae-Hyung Kim, Doo Young Kim.
